# Retrospect of the Medical Sciences

**Published:** 1843-04-29

**Authors:** 


### EMPLOYMENT OF MURIATE OF AMMONIA IN SCIRRHUS OF THE STOMACH.

The above salt having been administered by German practitioners with decided benefit in cases of induration and degenerescence of the bladder, prostate gland, &c., Trusen was induced to employ it in the following instances:

Case 1.—A man, thirty years of age, accustomed to a sedentary life, and the too great use of alcoholic liquors and strongly spiced aliments, had evidently contracted a scirrhous disease of the pyloric extremity of the stomach. For some months, vomiting, with violent heartburn, &c., had always come on from three to four hours after taking food. Ordered, ammon. hydrochl. fifteen grains every two hours, combined with extract of liquorice. In a short time digestion was performed better, and the appetite restored. Care and abstemiousness in diet were enjoined, and at the end of a six months' persistence in the use of the medicine, the vomitings ceased entirely. A clogged state of the stomach, from superabundance of mucus and habitual constipation, were removed by a six week's visit to the muriated-chalybeate springs of Cudova, and the man subsequently regained perfect health.

Case 2.—A man of age, and sedentary habits, similar to the foregoing, had been for some time affected with arthritis, &c., on the removal of which he began to be subject to an invariable rejection of the whole of his food a few hours after it had been taken, accompanied by atrophy and emaciation. He entered, however, upon the use of muriate of ammonia in drachm doses, (!) which he continued for seven months, when his vomitings had wholly and permanently disappeared.

The system of the patient soon adapted itself without inconvenience to the above large doses of the remedy, which were taken in infusion of ginger.—*Lond. Lan., from Hufeland's Journ.*

---

### STRUCTURE OF THE ARTERIES.

After a variety of conflicting and unsatisfactory accounts, Henle seems at length to have discerned such structures in the arteries as are adapted to the functions which experiment shows to be performed by them. His account of the general structure is briefly this:—

1st, They have an epithelial lining, consisting of a very thin layer of elliptic or rhombic lameller cells, which are sometimes elongated into longitudinal spindle-shaped fibres.*

2d, There is, immediately external to this, a layer of peculiar tissue, the *striated* or *fenestrated* coat, (corresponding to the *internal* coat of the older anatomists,) consisting of a very thin, rather stiff, and brittle membrane, often perforated by numerous round or oval apertures, and bearing pale, flat, very narrow fibres, which have, for the most part, a longitudinal direction, and give it a peculiar delicately-striated apeparance. This coat, which is often morbidly thickened, and when an artery is contracted, is commonly thrown into longitudinal folds, is produced by a metamorphosis of the epithelium, whose cells, as their nuclei disappear, coalesce and form a homogeneous membrane, on which the fibres are afterwards deposited, and which at last, as the apertures in it enlarge, is completely removed, leaving the fibres free.

3d, In some arteries there is, next, a coat formed by a single layer of *longitudinal* granular *fibres*, flat and tolerably wide, analogous to a coat which is much more prominent in the veins.

4th; A coat composed of *circular fibres*, (the *middle* or *elastic* coat of most foreign writers, the *muscular* coat of Hunter,) which forms the chief part of the arterial wall, and comprises all that can be torn from it in a transverse direction. Its fibres are flat, clear, and granular, and break with abrupt ends. Each of them is commonly marked along its middle by dots scattered, or regularly arranged in a longitudinal row, or by a narrow streak: these are the remains of elongated nuclei, which have formed, as it were, the pattern, according to which the homogeneous membrane in which they lay has broken up into the flat fibres. The streaks formed of the elongated nuclei often branch and anastomose, so as to form that kind of net-work which has led to this coat being mistaken for elastic tissue; whereas it is, in fact, the proper contractile coat of the artery, and is, in all respects of development and microscopic structure, similar to the layers of organic muscle in the stomach, &c.

5th, On its exterior there is a coat of genuine elastic tissue, (*tissu jaune*, the *elastic coat* of Hunter): this exists, however, only in the larger arteries; and its thickness, in comparison with that of the preceding, diminishes in direct proportion to the size of the artery. The direction of its fibres varies greatly in different arteries.

6th, The *external cellular coat*, consisting of common cellular tissue, with longitudinal closely-woven filaments.—*London Medical Gazette, from Report on the chief results obtained by the Microscope, in the study of Human Anatomy and Physiology.* By James Paget, Demonstrator, of Morbid Anatomy at St. Bartholomew's Hospital, &c.

* Remak and Reichert (Muller, Archiv. 1841, CLXXXVIII.) hold, that these are not the innermost cells of the vessels, but that within these, and in actual contact with the blood, there is a layer of flattened, round, and polyhedral cells, with round, yellowish nuclei and nucleoli. On all these observations by Henle, see Reichert's remarks.

### RETROSPECT OF THE MEDICAL SCIENCES.

INFLUENCE OF IMPROVED DIET ON THE HEALTH OF THE INSANE.

Before the revolution, the ordinary ration of bread for each lunatic in the Bicêtre was only a pound and a half. It was distributed in the morning; or, rather, it was instantaneously devoured, and part of the day was passed in a sort of hungry delirium. In 1792, the ration was increased to two pounds, and it was distributed in the morning, at noon, and in the evening, with a well-prepared soup. This is, no doubt, the reason of the difference of mortality found on making an abstract of the registers.

Out of 110 lunatics received into this asylum in 1784, fifty-seven died; that is to say, more than half. In 1788, the proportion was 93 to 151. On the contrary, during the second and third years of the republican era, the mortality was only an eighth of the whole number.—*Lond. Med. Gaz. from Traité Medico-Philosophique sur l' aliénation mentale.*

---

*On Fatty Degeneration of the Arteries, with a Note on some other Fatty Degenerations.* By GEORGE GULLIVER, F. R. S.

The author, remarking how vaguely the epithets, atheromatus, steatomatous, &c., have been applied by pathological writers to diseased arteries, and that the morbid deposit between the middle and inner coats, and in the substance of the former, has not, as far as he knows, yet been submitted to precise examination, gives the result of his own observations, from which it appears that the disease is really of a fatty nature. A microscopic examination of it brings into view a multitude of crystalline plates, fatty globules, with albuminous and earthy particles. Several specimens of the crystals were sent for examination to Dr. Davy, who ascertained that they are of cholesterine.

The fatty matter is easily extracted by boiling alcohol, and the crystals of cholesterine are seen to be deposited as the solution cools. The author has examined numerous specimens of the disease, and never failed to observe these crystals and the fatty globules in the deposit, and also generally in the substance of the altered middle coat. The microscopic characters are given in two figures.

The accuracy of Dr. Davy's observations (see his "Researches, Phys. and Anat.," vol. I., pp. 372 and 436) as to the thinning, &c., of the middle coat of the artery, is confirmed by Mr. Gulliver.

The importance of fatty degeneration of the coats of the arteries is insisted on, especially as to its general connection with thickening and puckering of the inner membrane, with aneurism, with obstruction, occlusion, or ossification of the vessels, and of those ruptures of them which are so frequently the cause of sudden death.

The author adds, that fatty degenerations are more common and of more importance than has yet been supposed. He mentions obstruction, by fatty particles, of the seminal tubes; and notices fatty degenerations of the blood, lungs, &c. The disease he describes as being more remarkable in "brown consolidation" of the lungs than in red consolidation; and these two diseases are described as affording distinct morbid products.—*Provincial Medical Journal*, March 18, 1843.

*A Normal and Abnormal Conscious State, alternating in the same Individual.* By JOHN WILSON, M. D., Physician to the Middlesex Hospital.

This case occurred in a boy, aged fourteen, a patient in the Middlesex Hospital, who is said to have complained of headache for two or three days, but whose appearance was healthy. For three or four days his appetite was inordinate, seizing upon any article of food he could meet with in the ward, though allowed full diet. When not eating or seeking for food he generally slept night and day. This abnormal state continued for three or four days, when he recovered his natural state of sleep, appetite, and consciousness. Then he had no recollection of what he had done, or of what had happened to him since his admission.

He was shortly discharged, but twice readmitted, each time presenting the same symptoms—*i. e.*, alternations of consciousness and unconsciousness.

No treatment was adopted. The author, for the present, reserves his opinion and inferences drawn from this case; his object is to invite further examination for similar cases; and when such occur then will be the time for discussion.—*Ibid.*

---

THE PROPORTION OF CARBONIC ACID FORMED IN TYPHUS.

Dr. Malcolm, of Belfast, has instituted a series of upwards of fifty experiments on the air expired by patients laboring under all types of the disease, and in all its stages. They were performed during the months of May and June, and invariably at one period of the day—viz., between the hours of eleven o'clock, a. m., and two o'clock, p. m., corresponding to the period when, according to Dr. Prout, the maximum proportion of carbonic acid gas is generated. Great care was taken in collecting the respired air, and in subjecting it afterwards to chemical analysis. The manner of breathing of the patient, the density of the atmosphere, temperature, age of the patient, and his mental state, were also carefully noted.

He concludes, from the results of his experiments, that in typhus fever the formation of carbonic acid gas during respiration is considerably less than in a state of health, the difference being, as nearly as possible, 1.5 per cent.—a difference which, in a number of cases, is much increased, and one too large to be the result of accidental circumstances. The quantity generated is least in the more severe forms of the disease. The diminished proportion, however, is not at all uniform, on some occasions the number being very low indeed, and in others rising to even as high as 2.7 per cent. The difference between the proportion of carbonic acid gas generally, as influenced by fever, and in the severe forms, is, it may be observed, as nearly as possible 2 per cent.—a difference, perhaps, too small to form the basis of any general conclusion.—*Ibid, from Lond. and Edin. Medical Journal*, Jan. 1843.

---

INTRODUCTION OF AIR IN THE VEINS.

The "Annales de la Chirurgie Française et Etrangère" contain a communication, by M. Marchal de Calvi, on the introduction of air into the veins, in the course of which he offers a new explanation of the cause of death produced thereby. The opinion that it depends on the distension of the heart, by which its contractions are prevented, is, he admits, supported by the experiments of Nysten with the different gases, but he says it does not explain those cases where the death is so frightfully sudden, as if the patient had been struck by lightning, and which resembles the immediate effect of a poisonous dose of prussic acid. In these cases there must be, he thinks, some toxic agent, and then arises the question, what is that agent? Hitherto to this there has not been offered any reply, but M. Marchal thinks he has found the answer, by discovering the presence of carbonic acid gas in the heart, which he is of opinion is disengaged every time air is introduced into it through the venous system. In this he is supported by the appearance of the blood in the pulmonary cavities of the heart, which, instead of being black, is red, it having been decarbonised by the contact with the atmospheric air, the oxygen of which has combined with its carbon, and formed the carbonic acid gas, to the action of which M. Marchal refers the death of the patient; and again, if, in performing experiments on this subject, the operator breathe through the tube into the vein, instead of injecting the purer air around him, the fatal effect will be much more rapidly induced, there being carbonic acid gas already generated in the injected fluid. The experiments of Nysten, however, with carbonic acid gas and oxide of carbon do not support this hypothesis.—*Ibid.*

ELECTRO-PUNCTURE.

M. Schuster has lately addressed a paper to the French Academy of Sciences, to prove that electricity is little serviceable in medicine unless it be applied through the means of acupuncture needles. Administered in this way, he asserts it may be employed with success against dropsies of all kinds, steatomatous and other tumours, engorgements and indurations, (and perhaps cancer,) goitre, varicose dilatations, chronic rheumatism, paralysis, amaurosis, and perhaps aneurism. Electro-puncture acts, he says, by directly stimulating the sensibility, contractility, and absorbent function; in the formation of internal eschars, and the detraction, piece by piece, of diseased growths; in decomposing the fluid contents of tumours; in procuring external openings for matter, provoking adhesive inflammations, &c.; though some of these effects seem capable of production by the needles alone, without the electricity.—*London Lancet.*

RENAL DISEASE SIMULATING CALCULUS IN THE BLADDER.

M. Segalas reports that he was called to attend on a child, from four to five years of age, which often placed its hands on the genitals, and experienced frequent desire to pass urine, which action, however, was always performed with pain. The urine was tolerably abundant, and yielded a muco-purulent deposit. The acuteness of the various symptoms had been considerably increased during some recent attacks of roseola and bronchitis. These symptoms apparently indicated the presence of a calculus in the bladder; M. Segalas, however, doubted that one really existed, having never met with this disease in any children but those of parents in a much lower sphere of life than those of this patient; and a careful examination proved that no urinary calculus in reality existed. Some time afterwards the child died of a cerebral affection, and the body was opened. The bladder, as well as the urethra, was found perfectly healthy and free from urinary deposit; but the left ureter was much dilated, and the pelvis of the corresponding kidney three or four times larger than that of the opposite side. The calices also were enlarged, and the cortical substance of the kidney was inflamed at several points. The right kidney was healthy.—*Ibid, from Gaz. de Hop.*

OBSERVATIONS ON IMPULSIVE INSANITY.

BY DR. MACKINNON.

Dr. Mackinnon, at the Medico-Chirurgical Society of Edinburgh, February 1st, 1843, remarked that insanity was occasionally displayed in an impulse to commit such acts as suicide, homicide, theft, fire-raising, &c., without any *delusion* of the understanding, in the common sense of the term, being present. Medical and legal writers, for a long period, exclusively regarded the intellectual faculties, when treating of mental derangement, and omitted all reference to the moral feelings—a part of the mental constitution at least equal in importance to the former. Thus monomania of the intellect was allowed, while monomania of the moral feelings was disregarded. The fact was, however, as remarked by Pritchard, that derangement of the moral feelings was fully more characteristic of insanity than delusion of the intellect. Among the inmates of an asylum, many are always found in whom this moral derangement exists in a greater degree than the intellectual. These prepare us for cases in which the derangement is *exclusively* in the moral part of the mental constitution. It certainly was sometimes a matter of extreme difficulty to distinguish such cases from others in which criminal acts were committed, for which the perpetrator was justly responsible. The best guides in the diagnosis were the existence or not of hereditary predisposition to mental disease, the presence or absence of the usual motives to crime, the consistency or inconsistency of the acts committed with the previous character of the individual, and, above all, the presence or absence of the physical signs of insanity, such as heat of head, injection of conjunctiva, furred tongue, and derangement of the cuticular and other secretions of the body. Dr. M. proceeded to give examples of impulsive insanity which had come under his observation. In the first case the *morale* was altogether deranged, and a disposition to commit both homicide and suicide exhibited, while the intellectual faculties, so far from being impaired, were above the average. Hereditary predisposition (a parent committed suicide), and the physical signs of disease were present. In the next case homicidal and suicidal propensities were felt, reasoned on, and deeply regretted by the patient, whose mind was of the highest order. In this case the physical signs of the disease were absent, and would have rendered it a matter of extreme difficulty to have sustained the plea of insanity if homicide had been committed. The whole history, however, conclusively showed a state of mental disease. In the next case the fire-raising and homicidal propensities were displayed. The physical signs were present. In the last case, detailed at length, a propensity to theft, and a wholly perverted *morale*, were exhibited. Dr. M. alluded to other cases of the same interesting, and, in a medico-legal point of view, most important form of insanity. —*Provincial Medical Journal, from Edinburgh Monthly Journal.*

